# Quality of Information and Future Directions. Comment on “Influence of Mass and Social Media on Psychobehavioral Responses Among Medical Students During the Downward Trend of COVID-19 in Fujian, China: Cross-Sectional Study”

**DOI:** 10.2196/23168

**Published:** 2021-02-18

**Authors:** Smriti Sasikumar, Hafsa Omer Sulaiman, Simran Bedi, Mikhail Nozdrin, Caroline Rundell, Sadia Zaman

**Affiliations:** 1 St George’s University of London Cranmer Terrace London United Kingdom; 2 King’s College London London United Kingdom; 3 Imperial College London United Kingdom; 4 University College London London United Kingdom

**Keywords:** COVID-19, social media, mass media, information quality

We read with great interest the recent article by Lin et al [1], which investigated the impact of mass and social media on psychobehavioral responses among medical students in China. We thank the authors for investigating this important issue in such a multifaceted manner. As medical students from four different medical schools in London, we questioned whether certain correlations discussed in the study could be examined further to elucidate the results and allow for some extrapolation. The results from this study are quite intriguing, and we believe this deeper analysis could spark other institutions around the world to examine the powerful effect of media on behaviors during a pandemic.

Since the study is based on Chinese media, we were unfamiliar with the quality of disseminated information related to COVID-19, particularly on social media. According to Ren et al [2], while Western media tends to center around controversy and individual experts, Chinese media sources focus on “mainstream positions” put forth by health agencies [3,4]. We suggest that an overview of how media sources are presented in China would be extremely useful in contextualizing the results of the study for non-Chinese audiences.

Furthermore, while the article’s findings suggest that exposure to mass media and social media could increase positive attitudes and reduce emotional response among university students, we believe that the credibility of the information on these platforms greatly contributes to the results. Misinformation and “fake news,” particularly on the internet, have become rampant [5]. The article presumes that the results are due to medical students being more discerning consumers of health information. While this may be possible, we suggest that analyzing the quality of the information consumed by the students, that is, whether the information was credible or not, would allow stronger correlations to be drawn as to why students had positive behavioral responses and lower trait anxiety.

This analysis on the quality of mass and social media could be done in a number of ways. For example, examining the quality of data could involve recruiting some participants from the current study to respond to health information of differing qualities. The information could be put into two categories: credible information and misinformation about COVID-19. Repeating the STAI-6 (Spielberger State-Trait Anxiety Inventory, 6 items) and the psychobehavioral response questionnaire on this controlled set of stimuli might yield more data on why mass and social media exposure reduced trait anxiety and changed behavior in the study population.

Lastly, we suggest that the data be further analyzed to examine whether the psychobehavioral and anxiety responses varied by age in the students. This suggestion stems from the observed trend that different age groups engage with different mass or social media platforms. Perhaps the quality and the type of mass or social media may have an effect on the observed results.

Implementing some of these suggestions could use the previously gathered data and provide a uniquely detailed viewpoint that could expand the results of the study. These additions and future steps could inspire cross-institutional collaboration, allowing us to better understand how media across the world can affect the behavior and anxiety levels of key populations during a pandemic.

## Abbreviations

STAI-6: Spielberger State-Trait Anxiety Inventory, 6 items
